# High pre-chemoradiotherapy pan-immune-inflammation value levels predict worse outcomes in patients with stage IIIB/C non-small-cell lung cancer

**DOI:** 10.1007/s12672-023-00851-8

**Published:** 2023-12-13

**Authors:** Erkan Topkan, Ahmet Kucuk, Emine Elif Ozkan, Duriye Ozturk, Ali Ayberk Besen, Huseyin Mertsoylu, Berrin Pehlivan, Ugur Selek

**Affiliations:** 1 Department of Radiation Oncology, Baskent University Medical Faculty, 01120 Adana, Turkey; 2 Clinic of Radiation Oncology, Mersin Education and Research Hospital, Mersin, Turkey; 3 Department of Radiation Oncology, Suleyman Demirel University, Isparta, Turkey; 4 Department of Radiation Oncology, Faculty of Medicine, Afyonkarahisar Health Sciences University, Afyonkarahisar, Turkey; 5 Department of Medical Oncology, Medical Park Hospital, Adana, Turkey; 6 Department of Medical Oncology, Istinye University, Istanbul, Turkey; 7 Department of Radiation Oncology, Bahcesehir University, Istanbul, Turkey; 8 Department of Radiation Oncology, Koc University School of Medicine, Istanbul, Turkey

**Keywords:** Non-small-cell lung cancer, Inflammation, Biological marker, Pan-immune-inflammation value, Prognosis survival

## Abstract

**Background and objectives:**

We explored the prognostic usefulness of the pan-immune-inflammation value (PIV) in patients with stage IIIB/C non-small-cell lung cancer (NSCLC) who underwent concurrent chemoradiotherapy (CCRT).

**Methods and patients:**

For all patients, the PIV was calculated using platelet (P), monocyte (M), neutrophil (N), and lymphocyte (L) measures obtained on the first day of CCRT: PIV = P × M × N ÷ L. Using receiver operating characteristic (ROC) curve analysis, we searched for the existence of an ideal cutoff that may partition patients into two groups with unique progression-free- (PFS) and overall survival (OS) results. The primary endpoint of this retrospective cohort research was to determine whether there were any significant relationships between pretreatment PIV measures and post-CCRT OS outcomes.

**Results:**

The present research included a total of 807 stage IIIB/C NSCLC patients. According to ROC curve analysis, the ideal PIV cutoff was 516 [area under the curve (AUC): 67.7%; sensitivity: 66.4%; specificity: 66.1%], which divided the whole cohort into two: low PIV (L-PIV: PIV < 516; N = 436) and high PIV (H-PIV: PIV ≥ 516; N = 371). The comparisons between the PIV groups indicated that either the median PFS (9.2 vs. 13.4 months; P < 0.001) or OS (16.7 vs. 32.7 months; P < 0.001) durations in the H-PIV group were substantially inferior to their L-PIV counterpart. Apart from the H-PIV (P < 0.001), the N_3_ nodal stage (P = 0.006), IIIC disease stage (P < 0.001), and receiving only one cycle of concurrent chemotherapy (P = 0.005) were also determined to be significant predictors of poor PFS (P < 0.05, for each) and OS (P < 0.05, for each) outcomes in univariate analysis. The multivariate analysis findings revealed that all four variables had independent negative impacts on PFS (P < 0.05, for each) and OS (P < 0.05, for each).

**Conclusions:**

The findings of this hypothesis-generating retrospective analysis claimed that the novel PIV was an independent and steadfast predictor of PFS and OS in stage IIIB/C NSCLC patients.

## Introduction

Approximately one-third of all non-small cell lung cancer (NSCLC) patients show a tumor or nodal involvement in the mediastinum at the time of diagnosis, indicating locally advanced disease [1]. Although the addition of post-chemoradiotherapy (CRT) immunotherapy is now considered the current care standard for medically fit locally advanced NSCLC (LA-NSCLC) patients following the publication of the favorable results of the phase 3 PACIFIC trial, immunotherapy is still challenging to achieve in many countries due to health insurance regulations [2]. Hence, definitive platinum-based concurrent CRT (CCRT) remains the current standard treatment for the vast majority of LA-NSCLC patients [3, 4]. Even following intensive CCRT, such patients' prognoses remain grim, with reported 5-year overall survival (OS) rates seldom surpassing 15% [5]. Clinical results of individuals at comparable LA-NSCLC stages after similar CCRT regimens can differ broadly. Such unanticipated and substantial outcome discrepancies might be attributed to the TNM (tumor-node-metastasis) staging system's sole dependence on the primary tumor's size and locoregional expansions, with no regard for tumor- and host-related biological variables [6]. Although this strategy is currently the best methodology for selecting the optimal therapy and estimating the outcomes of LA-NSCLC patients, such huge outcome variances underscore the need for additional factors to improve its prognostic and predictive abilities.

Systemic inflammation, the seventh cancer hallmark, has been consistently shown to play a crucial role in the genesis and progression of many solid tumors [7]. Because circulating immune-inflammatory cells such as platelets, monocytes, neutrophils, lymphocytes, and related cytokines exert a pivotal role in local and systemic immune and inflammatory responses, researchers have investigated the prognostic and predictive significance of these cells in LA-NSCLC patients, either alone [8, 9] or in their various blended forms [10, 11]. Whether the index was a single cell or a unique combination of cells [12, 13], accumulating evidence pointed to chronic systemic inflammation as a pivotal factor underlying disparities in LA-NSCLC prognoses following comparable treatment regimens [14, 15]. The pan-immune-inflammation value (PIV), which combines circulating platelets, monocytes, neutrophils, and lymphocytes, was recently developed by Fucà et al. as another comprehensive blood-borne biomarker [16]. Recent research indicates a clear link between pretreatment levels of PIV and patient outcomes in colorectal [16–18], breast [19–21], esophageal [22], small-cell and non-small-cell lung cancers [23, 24], Merkel cell carcinoma [25], and malignant melanomas [26, 27].

Despite data suggesting that the novel PIV is a robust predictor of treatment outcomes following various oncological therapies, most likely regardless of tumor type, PIV has never been questioned for its possible prognostic usefulness in stage IIIB/C NSCLC patients treated with radical CCRT. As a result, because the identification of novel biomarkers may be useful for more sophisticated prognostic stratification of such patients when used in conjunction with the TNM staging system, we conducted this retrospective cohort analysis to determine the prognostic significance of novel PIV in patients with stage IIIB/C NSCLC who had received definitive CCRT.Table: Kindly check and confirm the processed table layout for all tables.checked and confirmed

## Patients and methods

### Ethics, consent, and permissions

The retrospective research protocol was authorized by the Institutional Ethical Committee review board at Baskent University Medical Faculty before any data was collected. All participants provided written informed consent for the collection, analysis, and publication of blood samples and pathologic specimens, either personally or through legally licensed representatives. The Helsinki Declaration and Good Clinical Practice Guidelines and their amendments were followed throughout the study.

### Study population

An institutional retrospective database search was performed at Baskent University Medical Faculty’s Department of Radiation Oncology to select patients in stage IIIC (AJCC 8th ed.) who received CCRT with conventionally fractionated 60–66 Gy thoracic RT (2 Gy per fraction, 5 days a week) and at least one chemotherapy cycle concurrently between January 2010 and December 2020. To be qualified for the research, patients had to meet the following requirements: Eastern Cooperative Oncology Group (ECOG) performance score of 0–1; aged between 18 and 80 years; body mass index (BMI > 20.0 kg/m^2^); pathological proof for NSCLC [adenocarcinoma (AC) or squamous cell carcinoma (SCC)], stage IIIB/C disease by diagnostic computerized tomography (CT) and 18F-fluorodeoxyglucose positron emission tomography-CT (PET-CT), available pre-CCRT brain magnetic resonance imaging (MRI) scans; detailed chemotherapy and RT details; as well as complete blood count and biochemistry test results. Patients presenting with malignant pleural/pericardial effusion, involved contralateral supraclavicular lymph nodes, idiopathic pulmonary fibrosis, interstitial lung disease, a history of RT/chemotherapy, and insufficient pulmonary, cardiac, renal, or hepatic functions were ruled out of the research. Patients receiving steroid or anti-inflammatory medication, as well as immunosuppressive drugs, were also excluded to avoid any biasing effects on the results.

### Treatment details

All patients received intensity-modulated RT (IMRT) for the primary tumor site and involved lymph nodes, with elective nodal irradiation being prohibited. The IMRT plans were required to comply with our institutional clinical practice recommendations for newly diagnosed LA-NSCLC patients, which instruct the utilization of co-registered diagnostic CT and PET-CT scans. All target volume definitions, total and fractional dosage parameters, normal tissue tolerance dose limits, and concurrent chemotherapies were identical to those previously described [28]. In summary, all patients received 60–66 Gy of RT in 30–33 fractions (2 Gy per fraction) and 1–3 cycles of cisplatin/carboplatin plus one of docetaxel, paclitaxel, or vinorelbine concurrently. Supportive care measures were provided based on the needs of the patients.

### Pan-Immune-Inflammation value measurement

The pre-CCRT PIV was computed using Fucà's original formula [16]: PIV = P × M × N ÷ L, where P, M, N, and L represent pretreatment platelet, monocyte, neutrophil, and lymphocyte counts acquired on the first day of CCRT.

### Response assessment

Post-CCRT follow-up evaluations were scheduled every three months for the first 2 years, then every 6 months or more frequently as needed. Therapeutic response was assessed by complete blood count and biochemistry tests, PET-CT, or chest CT (following confirmation of complete metabolic response on PET-CT). The European Organization for Research and Treatment of Cancer (EORTC)-1999 guidelines were employed for this purpose. Supplementary radiologic and nuclear medicine imaging modalities were utilized only when there was a clinical suspicion of metastasis or when it was necessary to restage locoregionally recurrent NSCLC.

### Statistical methods

The association between the pretreatment PIV groups and overall survival (OS: time from the first day of CCRT to the date of death or the final visit) and progression-free survival (PFS) were the primary and secondary objectives of this retrospective cohort research (PFS: time from the first day of CCRT to the date of the first observation of disease progression or death or the final visit), respectively. The median and ranges were used to display continuous values, whereas percentage frequencies were used to describe categorical variables. We employed receiver operating characteristic (ROC) curve analysis to ascertain the ideal PIV cutoff, which might partition the study population into two groups with significantly different OS and PFS outcomes. The connection between PIV groups and other clinicopathological variables was investigated using the chi-square test, the Mann–Whitney U test, Student's t-tests, or Spearman correlations, as indicated. The Kaplan–Meier curves were used to plot survival curves, and the log-rank test was used to establish significance. Only the variables showing univariate significance were tested in the multivariate analysis to determine their independent prognostic value. Any two-sided P < 0.05 was considered significant. The Bonferroni correction was employed to minimize chance-related false-positive outcomes in comparative subgroup analyses involving three or more groups.

## Results

Table 1 shows the pretreatment patient and disease characteristics of 807 eligible stage IIIB/C NSCLC patients. There were 514 male (63.7%) and 293 female (36.3%) patients. Median age was 64 years (range: 26–79 years), with 232 (28.7%) being senior patients (> 70 years). The majority of patients had AC histology (N = 491; 60.8%) and stage IIIB disease (N = 433; 53.7%).

**Table 1:** Pretreatment patient and disease characteristics at presentation

Covariate	All patients (N = 807)	PIV < 516 (N = 436)	PIV ≥ 516 (N = 371)	P-value
Median age, y (range)	64 (26–79)	65 (29–79)	63 (32–79)	0.86
Age group, y (%)				0.63
≤ 70 years	575 (71.3)	315 (72.2)	260 (70.1)	
> 70 years	232 (28.7)	121(27.8)	111 (29.9)	
Gender, n (%)				0.72
Female	293 (36.3)	149 (34.2)	144 (38.8)	
Male	514 (63.7)	287 (65.8)	227 (61.2)	
ECOG, n (%)				0.66
0	214 (26.5)	110 (25.2)	104 (28.0)	
1	593 (73.5)	326 (74.8)	267 (72.0)	
Histology, n (%)				0.27
SCC	316 (39.2)	166 (38.1)	150 (40.4)	
AC	491 (60.8)	270 (61.9)	221 (59.6)	
T-stage, n (%)				0.39
1–2	179 (22.2)	109 (25.0)	70 (18.9)	
3–4	628 (77.8)	327 (75.0)	301 (81.1)	
N-stage, n (%)				0.009
2	348 (43.1)	208 (47.7)	140 (37.7)	
3	459 (56.9)	228 (52.3)	231 (62.3)	
Stage, n (%)				0.002
IIIB	433 (53.7)	257 (58.9)	176 (47.4)	
IIIC	374 (46.3)	179 (41.1)	195 (52.6)	

*PIV* Pan-immune-inflammation value, *ECOG* Eastern Cooperative Oncology Group, *SCC* Squamous-cell carcinoma, *AC* Adenocarcinoma, *T-stage* Tumor stage, *N-stage* Nodal stage

The median follow-up duration was 26.4 months (range: 2.1–137.3 months) for the whole study cohort. Two hundred ninety-four (36.4%) patients were still alive during this analysis, and 139 (17.2%) patients were disease-free. Thoracic RT doses were 60 Gy and 66 Gy in 62.7% and 37.3% of patients, respectively, while 184 (22.8%), 406 (50.3%), and 217 (26.9%) of patients were able to receive 1, 2, and 3 cycles of concurrent chemotherapy. Two hundred twenty-one (27.4%) patients received maintenance chemotherapy at the discretion of their treating medical oncologist (Table 2). The treatment was reasonably well tolerated, with 33.1% and 8.3% acute and late grade 3–4 toxicity rates, respectively. The prevailing acute grade 3–4 toxicities seen in this study were esophagitis (n = 97; 12.0%)) and refractory nausea/vomiting (n = 86; 10.7%). Meanwhile, the most often occurring late grade 3–4 toxicities were pneumonitis/pulmonary fibrosis (n = 23; 2.9%) and peripheral neuropathies (n = 21; 2.6%). Although there were no toxicity related deaths during the acute phase of the treatment, but 6 (%) patients died due late toxicities: trachea-esophageal fistula (n = 3; 0.37%), fatal pulmonary hemoptysis (n = 2; 0.24%), and pulmonary fibrosis (n = 1; 0.12) (Table 2).

**Table 2:** Treatment characteristics, toxicity outcomes, and survival results for the entire research cohort and per pan-immune-inflammation value groups

Outcome	All patients (N = 807)	PIV < 516 (N = 436)	PIV ≥ 516 (N = 371)	P-value
IMRT dose, n (%)				0.83
60 Gy	301 (37.3)	157 (36.0)	144 (38.8)	
66 Gy	506 (62.7)	279 (64.0)	227 (61.2)	
Concurrent Ctx cycles, n (%)				0.27
1	184 (22.8)	85 (19.5)	99 (26.7)	
2	406 (50.3)	226 (51.8)	180 (48.5)	
3	217 (26.9)	125 (28.7)	92 (24.8)	
Maintenance Ctx, n (%)				0.22
Absent	586 (72.6)	302 (69.3)	284 (76.5)	
Present	221 (27.4)	134 (30.7)	87 (23.5)	
Acute Grade 3–4 toxicity, n (%)				0.007
Absent	540 (66.9)	317 (72.7)	223 (60.1)	
Present	267 (33.1)	119 (26.3)	148 (39.9)	
Late Grade 3–4 toxicity, n (%)				0.032
Absent	740 (91.7)	408 (93.6)	332 (89.5)	
Present	67 (8.3)	28 (6.4)	39 (10.5)	
Grade 5 toxicity, n (%)				0.74
Absent	801 (99.3)	434 (99.5)	367 (98.9)	
Present	6 (0.7)	2 (0.5)	4 (1.1)	
DM rate, n (%)				0.002
Absent	139 (17.2)	101 (23.2)	38 (10.2)	
Present	668 (82.8)	335 (76.8)	333 (89.8)	
≤ 6 months	192 (28.7)	66 (19.7)	126 (37.8)	
6–12 months	208 (31.1)	109 (32.5)	99 (26.7)	
> 12 months	268 (40.2)	160 (47.8)	108 (35.5)	
PFS				< 0.001
Median, mo	12.2	13.4	9.2	
5-year (%)	10.1	15.6	4.6	
8-year (%)	9.7	14.3	0	
OS				< 0.001
Median, mo	23.5	32.7	16.7	
5-year (%)	16.9	26.8	5.6	
8-year (%)	12.8	21.4	0	

*PIV* Pan-immune-inflammation value, *IMRT* Intensity-modulated radiotherapy, *Ctx* Chemotherapy, *DM* Distant metastasis, *PFS* Progression-free survival, *OS* Overall survival, *mo.* months

The survival analysis for the total research cohort revealed that the median, 5-year, and 10-year PFS rates were 12.2 months [95% confidence interval (CI): 11.6–12.8 months], 10.7%, and 9.1%, respectively. Whereas, the corresponding OS rates were 23.5 months (95% CI 21.5–25.5 months), 16.9%, and 12.8%. During the last visit, locoregional control was achieved in 254 (31.5%) patients, while the DM-free rate was 21.2% (N = 171). Isolated locoregional recurrences were reported only in 33 (4.1%) cases.

**Fig. 1:**
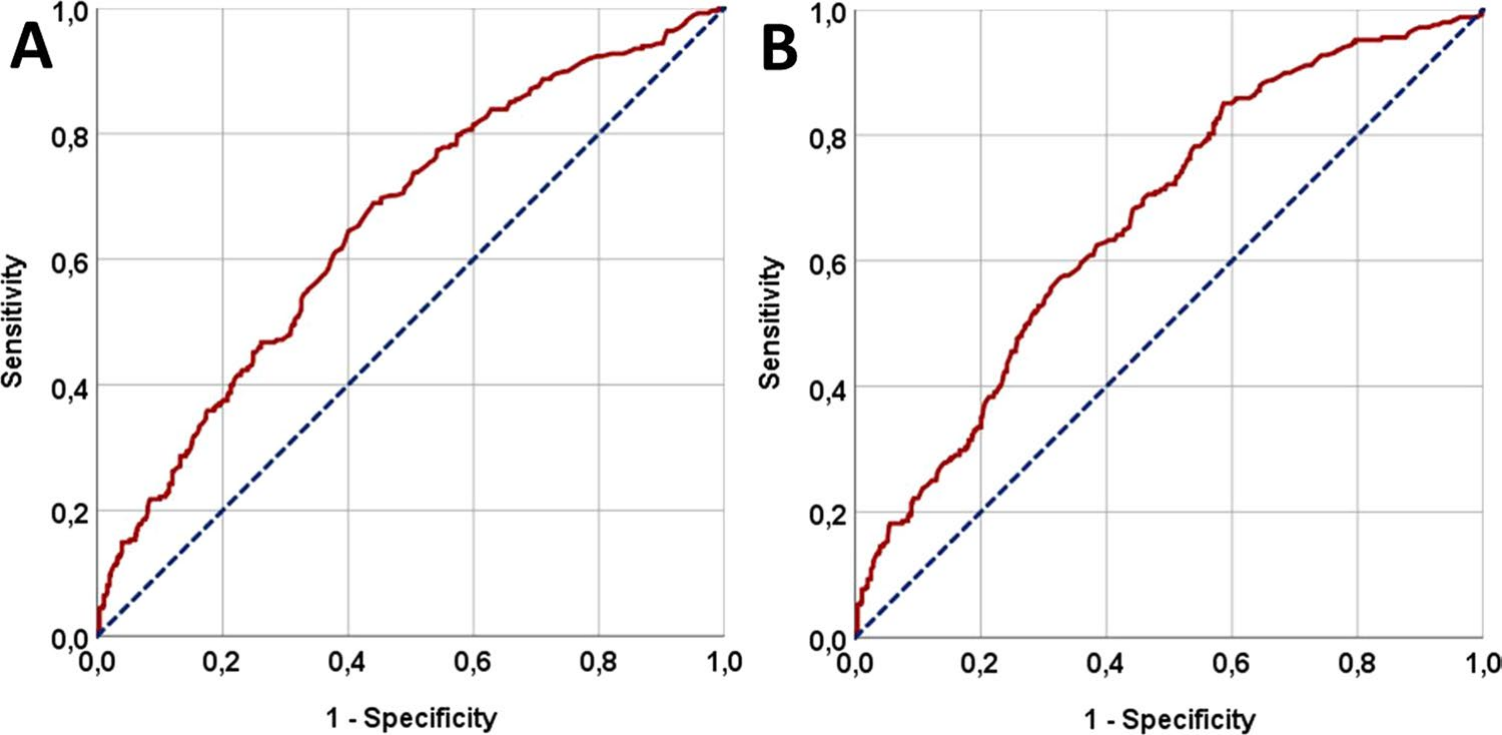
The results of receiver operating characteristic (ROC) curve analyses: **A** Progression-free survival. **B** Overall survival

**Fig. 2:**
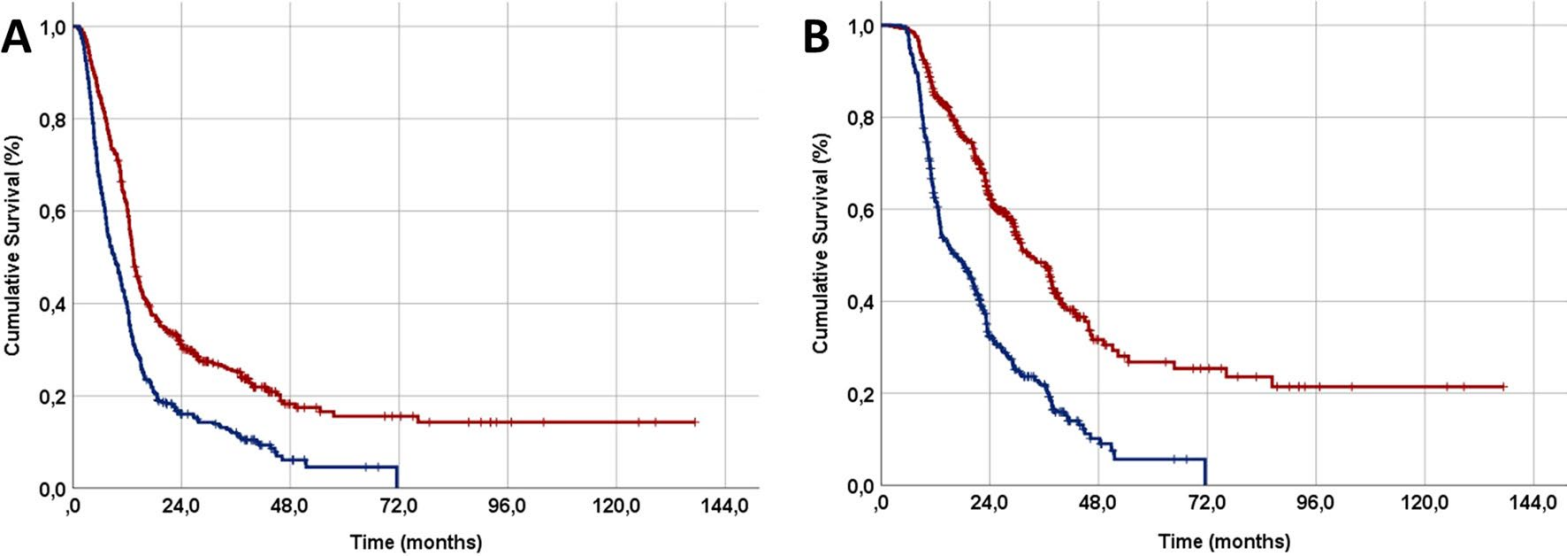
Comparative survival outcomes between the pan-immune-inflammation value (PIV) groups. **A** Progression-free survival. **B** Overall survival (red: low pan-immune-inflammation value; dark blue: high pan-immune-inflammation value)

The optimal cut-off values for PIV using ROC curve analysis for PFS and OS were determined at 518 [area under the curve (AUC): 66.9%; sensitivity: 66.2%; specificity: 65.8%] and 516 (AUC: 67.7%; sensitivity: 66.4%; specificity: 66.1%) points (Fig. 1). We adopted 516 of OS as the common cutoff value for subsequent PFS and OS comparisons because both cutoffs were nearly equivalent. Overall, 436 patients (54.0%) had a low PIV (L-PIV: PIV < 516), whereas 371 (46%) had a high PIV (H-PIV: PIV ≥ 516). Results of the comparative analyses between the two PIV groups showed that the H-PIV patients had significantly shorter median PFS (9.2 vs. 13.4 months; P < 0.001) and OS (16.7 vs. 32.7 months; P < 0.001) durations than their L-PIV counterparts (Fig. 2 and Table 2). Indicating that the higher PIV's adverse effects were sustained over time, the H-PIV group also had poorer actuarial 5-year and 8-year PFS and OS rates (Table 2). Considering the toxicities of acute and late treatment, it was found that there were no differences in grade 5 toxicity rates between the two PIV groups (P = 0.74). However, patients in the H-PIV group experienced significantly more acute (39.9% vs. 26.3%; P = 0.007) and late (10.5% vs. 6.4%; P = 0.032) grade 3–4 toxicities compared to their L-PIV counterparts (Table 2).

In univariate analysis, the additional characteristics that predicted poorer PFS (P < 0.05 for each) and OS (P < 0.05 for each) outcomes were the N_3_ nodal stage (vs. N_2_), IIIC stage (vs. IIIB), and 1 concurrent chemotherapy cycle (vs. 2–3) (Table 3). However, in multivariate analysis (Table 3), stage IIIC, 1 concurrent chemotherapy cycles, and the H-PIV group retained their independent significance as markers of diminished PFS (P < 0.05 for each) and OS (P < 0.05 for each), but N-stage lost its relevance (P = 0.14) (Table 3).

**Table 3:** Results of univariate and multivariate analyses

Characteristic	Patients (N)	Median PFS (months)	Univariate P- value	Mulivariate P- value	HR (95% CI)	Median OS (months)	Univariate P-value	Mulivariate P- value	HR (95% CI)
Age group			0.33	–	–		0.29	–	–
≤ 70 years	466	12.7				24.3			
> 70 years	185	11.3				22.4			
Gender			0.41	–	–		0.35	–	–
Female	224	11.6				22.8			
Male	427	12.7				24.7			
ECOG			0.85	–	–		0.89	–	–
0	486	12.4				23.8			
1	165	11.9				23.2			
Histology			0.64	–	–		0.37	–	–
SCC	244	11.6				22.2			
AC	407	12.8				23.8			
T-stage			0.17	–	–		0.15	–	–
1–2	228	13.4				24.8			
3–4	423	11.7				22.3			
N-stage			0.004	0.006	0.74 (0.57–0.93)		0.004	0.014	0.62 (0.45–0.81)
2	348	14.8				28.6			
3	459	11.3				20.1			
Stage			< 0.001	< 0.001	0.79 (0.70–0.89)		< 0.001	< 0.001	0.66 (0.51–0.83)
IIIB	433	13.2				29.3			
IIIC	374	10.9				20.8			
IMRT dose			0.92	–	–		0.95	–	–
60 Gy	301	12.4				23.7			
66 Gy	506	12.1				23.4			
Concurrent Ctx cycles			0.002	0.005	1.21 (1.04–1.41)		0.001	0.003	1.23 (1.07–1.39)
1	184	10.9				21.4			
2–3	623	13.3				26.7			
Maintenance Ctx			0.79	–	–		0.86	–	–
Absent	586	12.5				24.2			
Present	221	11.9				23.1			
PIV group			< 0.001	< 0.001	0.53 (0.39–0.68)		< 0.001	< 0.001	0.47 (0.36–0.59)
< 516	436	13.4				32.7			
≥ 516	371	9.2				16.7			

*PFS* Progression-free survival, *OS* Overall survival, *HR* Hazard ratio, *CI* Confidence interval, *ECOG* Eastern Cooperative Oncology Group, *SCC* Squamous cell carcinoma, *AC* Adenocarcinoma, *T-stage* Tumor stage, *N-stage* Nodal stage, *IMRT* Intensity-modulated radiotherapy, *Ctx* Chemotherapy, *PIV* Pan-immune-inflammation value

**Fig. 3:**
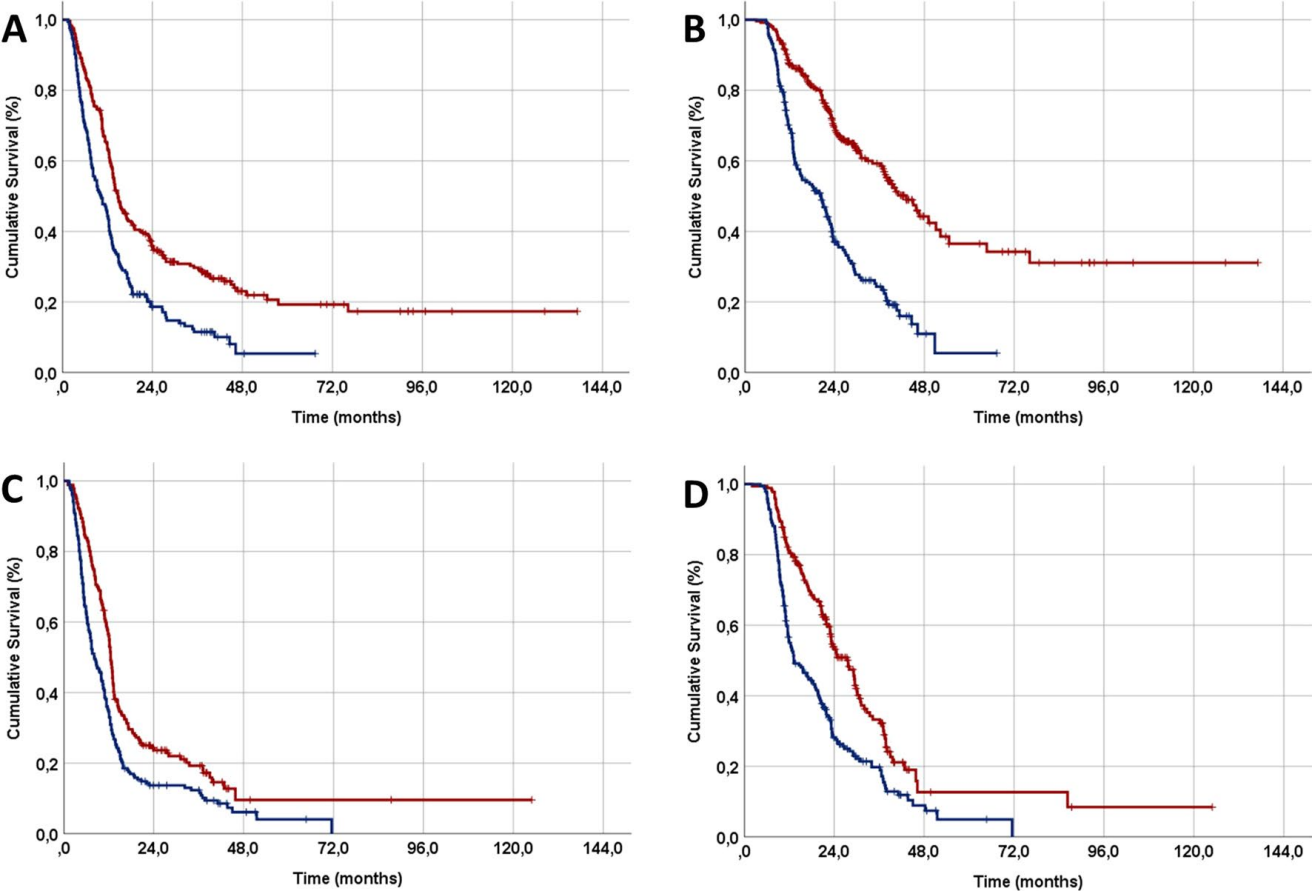
Subgroup survival analyses outcomes of stages IIIB and IIIC patients’ per pan-immune-inflammation value (PIV) status. **A** Progression-free survival for stage IIIB patients, **B** Overall survival for stage IIIB patients, **C** Progression-free survival for stage IIIC patients, and **D** Overall survival for stage IIIC patients (red: low pan-immune-inflammation value; dark blue: high pan-immune-inflammation value)

To determine the relative impact of the PIV grouping on the separate disease stages, we performed survival analyses according to these variables, using a Bonferroni corrected P-value of < 0.012 for significance. As presented in Fig. 3 and Table 4, patients with H-PIV had substantially inferior PFS and OS rates than their L-PIV counterparts in either of the stages IIIB or IIIC patients (P < 0.001 for each). Remarkably, the PFS and OS outcomes for patients in stage IIIB and IIIC of H-PIV were notably unfavorable, indicating a significant detrimental effect of heightened systemic inflammation in both disease stages. This observation suggests that incorporating systemic inflammation as an adjuvant to disease staging might be beneficial in further stratification of such patients (Fig. 3 and Table 4).

**Table 4:** Survival outcomes for stage IIIB and IIIC patients per their pan-immune-inflammation value status

Endpoint	L-PIV	H-PIV	P-value
Stage IIIB			
Median PFS, mo. (95% CI)	17.9 (15.1–20.7)	10.2 (8.3–12.1)	< 0.001
5-year PFS, %	19.8	4.4	
Median OS, mo. (95% CI)	46.7 (42.6–50.8)	18.7 (16.6–20.8)	< 0.001
5-year OS, %	38.3	4.7	
Stage IIIC			
Median PFS, mo. (95% CI)	14.8 (12.5–17.1)	8.1 (6.6–9.6)	< 0.001
5-year PFS, %	11.1	3.6	
Median OS, mo. (95% CI)	27.6 (23.4–31.8)	13.8 (11.6–16.0)	< 0.001
5-year OS, %	16.7	3.8	

*H-PIV* High pan-immune-inflammation value, *L-PIV* Low pan-immune-inflammation value, *PFS* Progression-free survival, *OS* Overall survival, *mo.* months, *CI* Confidence interval

## Discussion

Even after equivalent oncological therapy, genetic and biological variables such as systemic immunological and inflammatory states might result in drastically divergent outcomes in stage IIIB/C NSCLC patients, highlighting the need for novel biomarkers for better prognostic stratification of such patients. Hence, the present research aimed to investigate the prognostic usefulness of the novel PIV in stage IIIB/C NSCLC patients who underwent definitive CCRT at our institution. The following were the two most important conclusions from the current retrospective cohort analysis: First, indicating prognostic merit, the pre-CCRT PIV was able to independently stratify stage IIIB/C patients into two groups with significantly different PFS (P < 0.001) and OS (P < 0.001) results irrespective of the disease stage. Second, in the H-PIV patient group, the PFS and OS results of stage IIIB and IIIC patients were practically identical. This finding strongly implies that aggravated systemic inflammation is a crucial factor in worsening treatment outcomes, which could complement the prognostic power of the current staging system.

Chronic systemic inflammation, which plays a crucial role in the genesis, development, and progression of cancers, has been nominated as the seventh cancer hallmark [29, 30]. According to accumulating study findings, the prognosis of cancer patients is intricately bound to host-related characteristics. One of these factors is the chronic systemic inflammatory response, which increases tumor angiogenesis, inhibits antitumor immunity, enables immune response escape, accelerates the metastatic process, and develops resistance to anticancer treatments such as chemotherapy and radiation therapy [31]. A practical way of determining the intensity of the systemic inflammatory response in solid cancers is to measure the acute-phase proteins such as albumin and C-reactive protein and the blood-borne cellular markers like monocytes, neutrophils, platelets, and lymphocytes. Past research has established the prognostic utility of generic systemic inflammation markers such as neutrophil-to-lymphocyte ratio (NLR), platelet-to-lymphocyte ratio (PLR), lymphocyte-to-monocyte ratio (LMR), systemic immune-inflammation index (SII), systemic inflammation response index (SIRI), and advanced lung cancer inflammation index in NSCLC patients undergoing various cancer therapies [32–34]. But, interestingly, forming a rational ground for this present first attempt, despite the availability of appealing results from various studies and a meta-analysis indicating a strong link between pretreatment levels of PIV and patient outcomes in many solid tumors [27], the prognostic value of PIV has never been investigated in stage IIIB/C NSCLC patients treated with radical C-CRT before.

The discovery of the novel PIV as an independent prognostic factor that uniquely stratifies stage IIIB/C NSCLC patients into two significantly divergent PFS and OS groups following concurrent CCRT was the principal contribution of our current research to the NSCLC literature. Accordingly, the patients presenting with an H-PIV had significantly shorter median PFS (9.2 vs. 13.4 months; P < 0.001) and OS (16.7 vs. 32.7 months; P < 0.001) durations compared to their L-PIV counterparts, which appears to be just marginally better than the matching median survival times of metastatic NSCLC patients. Despite recent research proposing that PIV has a poor prognostic value in colorectal, breast, esophageal, and small-cell lung cancers, Merkel cell carcinoma, and malignant melanomas after various oncological therapies [27], it is difficult for us to explain the exact reason for our findings in the absence of similarly designed LA-NSCLC studies. Still, by thoroughly considering the well-established vital roles of local and systemic inflammation in all stages of tumor genesis and malignant progression, as well as the inflammatory cell ingredients of the unique PIV formula: monocytes, platelets, neutrophils, and lymphocytes, some reasonable conclusions can be drawn on this pressing issue. First, our present findings are consistent with previous PIV findings in diverse tumor primaries [27], suggesting that the unique PIV's predictive value is driven by the immunological and inflammatory condition of the afflicted host rather than the kind of tumor primary. Confirming this suggestion, the results of a recent meta-analysis involving 15 studies and 4942 patients revealed that patients with higher PIV levels had a significantly higher risk of disease progression (P < 0.001) and mortality (P < 0.001) than those with lower PIV levels regardless of disease stage or treatment choices [27]. Second, worsened chronic inflammation may have raised treatment-related acute and subacute severe toxicities, lowering treatment tolerance and likely contributing to worse results. Ascertaining this assumption, the H-PIV patients exhibited higher grade 3–4 acute toxicity rates than the corresponding L-PIV patients (39.9% vs. 60.1%) (P = 0.007). Similarly, while not statistically significant, the percentage of H-PIV patients who could undergo two or three rounds of concurrent chemotherapy was numerically lower (73.3% vs. 80.5% for L-PIV patients; P = 0.27). Third, given the critical roles of monocytes, neutrophils, and platelets in every stage of carcinogenesis, increased counts of these pro-inflammatory cells and/or a companion decrease in lymphocyte counts may indicate a suppressed antitumor immune response and an aggravated inflammatory response, which facilitates tumor growth, invasiveness, metastasis, and immune system escape, eventually leading to poor tumor control and survival rates [31]. And fourth, alternatively, our findings may be viewed as unsurprising, given that innovative PIV is a fusion of SII and SIRI, both of which have demonstrated prognostic utility in patients with locally advanced NSCLC treated with definitive CCRT [33, 34]. Although more research is needed to elucidate the precise and probably more complex mechanism(s), the cumulative effect of decreased immunogenic lymphocytes and increased immunosuppressive monocytes, platelets, and neutrophils may be fully accountable for the deteriorated PFS and OS results in the H-PIV patient group, as we have seen here.

An unexpected but equally important finding of our present research was that, although PFS and OS results of stage IIIB and IIIC patients were significantly different in L-PIV patients, corresponding results were statistically indistinguishable in H-PIV patients. This discovery is particularly significant since it may imply that the present TNM staging system's discriminative power is compromised in terms of accurately predicting the prognosis of stage IIIB and IIIC NSCLC patients presenting with H-PIV. However, apart from the TNM staging system's inherent weaknesses, this finding may also imply that the chronically exacerbated systemic inflammatory conditions may induce the emergence of radio- and or chemoresistant clones with a highly aggressive tumor phenotype that has a high potential for early locoregional recurrences and widespread distant DMs, resulting in an unpredictably poor prognosis in such patients [35]. Confirming these conclusions, the median PFS of H-PIV stage IIIB and IIIC patients were only 10.2 and 8.1 months, respectively, resembling the metastatic NSCLC research results [36]. Therefore, it is plausible to assume that the majority of H-PIV patients had latent DMs prior to starting CCRT that were not only undetected by current staging methods but also resistant to systemic chemotherapeutics. If our findings and assumptions are supported by additional research, a combination of TNM subgroups and novel PIV could be used to more accurately predict the prognosis of stage IIIB/C NSCLC patients undergoing radical CCRT, as such a combined approach could reflect both tumor characteristics and the host’s systemic immune and inflammatory status at the same time.

There are certain limitations to the current research: First, because this was a single-center retrospective cohort investigation, the data provided here are vulnerable to sampling bias. Second, despite the fact that PIV is a dynamic biomarker that can fluctuate drastically during and after CCRT due to the variations in host immunity, systemic inflammatory response status, and tumor load, we grounded our findings on a single time point snapshot of pre-CCRT PIV readings. However, the PIV variations may also imply tumor response or progression much earlier than the emergence of conspicuous radiographic abnormalities at any particular time. As a result, future research should concentrate on PIV dynamics to define a more meaningful cutoff, if one exists, that might aid usefully in the more dependable prognostic stratification of such patient groups. And third, we did not look into any potential links between the PIV groups and other immunological and inflammatory markers, such as the cytokines and chemokines produced and secreted by the unique PIV components. As a result, we may have missed an opportunity to reveal the specific processes behind the likely link between PIV measurements and survival outcomes in stage IIIB/C NSCLC patients. Finally, although our study suggests that the PIV could be a reputable, practical, factually quantifiable, reproducible, and affordable novel prognostic index for stage IIIB/C NSCLC patients undergoing radical CCRT, it is essential to note that our findings are not conclusive and should be considered hypothesis-generating rather than firm recommendations until more extensive studies use PIV as a stratification factor.

## Conclusions

Although more research is needed to ascertain its prognostic value, the findings of this hypothesis-generating retrospective study indicated that the novel PIV was an independent and dedicated predictor of PFS and OS outcomes in patients with stage IIIB/C NSCLC, which may complement the prognostic significance of the existing staging system.

## Data Availability

The datasets used and/or analyzed during the current investigation are available from the Baskent University Department of Radiation Oncology Institutional Data Access: adanabaskent@baskent.edu.tr for researchers who meet the requirements for access to sensitive data.
